# Genetic Monitoring of a Newly Established Grey Wolf Population in a Peri-Urban Protected Area with First Insights into Wolf–Dog Hybridization in Greece

**DOI:** 10.3390/genes17030278

**Published:** 2026-02-27

**Authors:** Aimilia Ioakeimidou, Yorgos Iliopoulos, Aristotelis Moulistanos, Kerasia Galani, Athanasia Fyta, Eirini Antoniadi, Georgios Bartzokas, Theodoros Kampouris, Caroline Sophie Birkenhain, Gregor Rolshausen, Carsten Nowak, Triantafyllos Akriotis, Maria Papandreou, Nikoleta Karaiskou

**Affiliations:** 1 Department of Environment, School of the Environment, University of the Aegean, 81100 Mytilene, Greece; aimiliaioa@gmail.com (A.I.); takr@aegean.gr (T.A.); 2 Callisto Wildlife and Nature Conservation Society, 54640 Thessaloniki, Greece; yiliop2@gmail.com (Y.I.); eiriniant@biol.uoa.gr (E.A.); georgios.bartzokas@gmail.com (G.B.); 3 Department of Genetics, Development and Molecular Biology, School of Biology, Aristotle University of Thessaloniki, 54124 Thessaloniki, Greece; amoulist@bio.auth.gr (A.M.); kerasiag@bio.auth.gr (K.G.); athafyta@bio.auth.gr (A.F.); 4 Intermunicipal Stray Animals Care Centre, Schisto Industrial Park, 18863 Perama, Greece; theodoroskampouris91@gmail.com; 5 Centre for Wildlife Genetics, Senckenberg Research Institute and Natural History Museum Frankfurt, Clamecystrasse 12, 63571 Gelnhausen, Germany; caroline.birkenhain@senckenberg.de (C.S.B.); gregor.rolshausen@senckenberg.de (G.R.); carsten.nowak@senckenberg.de (C.N.); 6 Institute for Ecology, Evolution and Diversity, Faculty of Biological Sciences, Goethe University Frankfurt, Max-von-Laue-Straße 13, 60438 Frankfurt am Main, Germany; 7 Management Unit of Parnitha and Schinias National Parks and Protected Areas of Saronikos Gulf, Natural Environment and Climate Change Agency (N.E.C.C.A.), 13672 Acharnes, Greece; m.papandreou@necca.gov.gr; 8 Genomics and Epigenomics Translational Research (GENeTres), Centre for Interdisciplinary Research and Innovation (CIRI-AUTH), Balkan Centre, 57001 Thessaloniki, Greece

**Keywords:** large carnivores, anthropogenic hybridization, microsatellites, non-invasive sampling, genetic diversity

## Abstract

Background/Objectives: Following centuries of systematic eradication, grey wolf (*Canis lupus*) populations across Europe have experienced a significant recovery over recent decades, which leads to concerns regarding, among others, anthropogenic hybridization. In Greece, the genetic status of the wolf population is largely unknown to date. Here, we genetically monitor and test for wolf–dog hybridization events in a recently established wolf population in the Parnitha Protected Area, in close vicinity to the capital city of Greece. Methods: One hundred and twenty-four wolf scat samples were genotyped at 20 canine-specific autosomal microsatellite loci and compared to available reference tissue samples from wolves and free-ranging dogs. Results: A minimum of 31 unique wolf individuals were identified, structured into at least three packs. No wolf–dog hybrids were detected in the study area. To validate the accuracy of the microsatellite analysis, an ancestry informative 93-SNP panel was applied to non-invasive wolf DNA samples from the study area, confirming the absence of hybrids among them. However, a possible wolf–dog hybrid was detected among reference wolf samples collected in Northern Greece, where individuals with atypical morphological traits are observed. The estimated census population size was in accordance with concurrently obtained camera trapping data, while heterozygosity values were low. Conclusions: This research represents the first systematic effort in Greece to genetically monitor wolves recently established in a protected area. It highlights the need for targeted management strategies based on genetic data to ensure balanced long-term conservation of wolves in peri-urban areas.

## 1. Introduction

The natural recovery of large predators in Europe during the last half-century is primarily attributed to legal protection of their habitats and their populations against degradation and poaching [1]. This expansion is not restricted to protected areas but extends significantly beyond them to cultural landscapes. Inevitably, wolves and other large carnivores colonize new habitats dominated by human land use as their individuals disperse. This expansion is associated with an increase in wolf–livestock conflict [2] and may also be related to attacks on hunting and free-ranging dogs [3].

In view of massive public interest and strict requirements from EU-wide and national conservation legislation over the past centuries, multiple approaches have been developed to provide population size estimates [4]. Estimations of wolf abundance by combining standard field methods such as camera trapping, howling detection or trapping questionnaires lack credibility and accuracy due to the difficulty in identifying individuals [5]. Thus, non-invasive survey methods based on DNA extracted from field-collected samples (e.g., scats, hairs, urine) are increasingly used together with capture–recapture (CR) models to accurately assess demographic parameters of wolf populations [6,7].

Non-invasive genetic methods have also proven effective when aiming to identify anthropogenic hybridization events between grey wolf and domestic dog (*C. familiaris*). The genetic identification of putative hybrids is essential as their atypical morphological traits alone are not considered reliable evidence of hybridization [8]. Wolf and domestic dog share a recent evolutionary history as their ancestors diverged approximately 40–14 kya ago [9]. Although these two taxa are closely related, they differ significantly in behavior and morphology [10,11]. Wolf–dog hybridization (WDH) may affect the ecological and genetic integrity of the wolf in the long term, including its adaptive capacity [12]. Although accurate European-wide assessments of WDH are lacking to date, available data suggest that Southern and Eastern European wolf populations are more vulnerable to genetic introgression from dogs due to the large numbers of free-ranging dogs in those regions [13].

Greece, which represents one of Europe’s southernmost edges of wolf distribution, currently hosts a relatively large population estimated at a minimum of 2075 wolves and consisting of at least 255 packs [14]. While wolves are primarily distributed across the northern and central Greek mainland, recent records of a nine-member pack have also confirmed the species’ expansion in the south, namely the Peloponnese.

Although wolves in Greece experienced major declines until the mid-twentieth century like elsewhere in Europe, they remained locally abundant and a part of Greek biodiversity throughout the centuries. Until the 1980s, wolf distribution was limited to the northern mainland, covering only half of its current range. The subsequent recovery of the wolf population in Greece was largely facilitated by the adoption of key international conventions into national legislation, which led to the reduced use of poisoned baits and the prohibition of wolf hunting [15].

Since the 1990s, the wolf population has begun to expand into Central Greece, eventually recolonizing the Mount Parnitha area. Parnitha is a forested mountainous area (maximum altitude: 1413 m.a.s.l.), with its peak located only 30 km from the center of Athens and its southern flanks overlapping the outskirts of the city [16]. It constitutes a biodiversity hotspot in Southern Europe, with remarkably rich flora, fauna and high endemism (e.g., 1093 plant species, 42 mammal species, and 93 Greek endemic plant species) [16]. Among its fauna, red deer (*Cervus elaphus*) stands out as the most important remnant of the wild Greek population, which has been severely affected by wildfires in recent decades [17] and by wolf re-establishment in Mount Parnitha since 2014 [18]. According to the most recent estimate (2017), the minimum population size was 21 individuals living in two packs [19].

The aim of the present study was to shed light on the population structure of wolves in a recently recolonized area of Central Greece, after an absence of at least 60 years [18], e.g., to test (a) whether wolves have already formed reproductive packs or are mostly dispersers and (b) whether close vicinity to human settlements could increase hybridization events. Moreover, we aimed at assessing levels of genetic diversity and population density in the area.

## 2. Materials and Methods

### 2.1. Sample Collection

**Figure 1:**
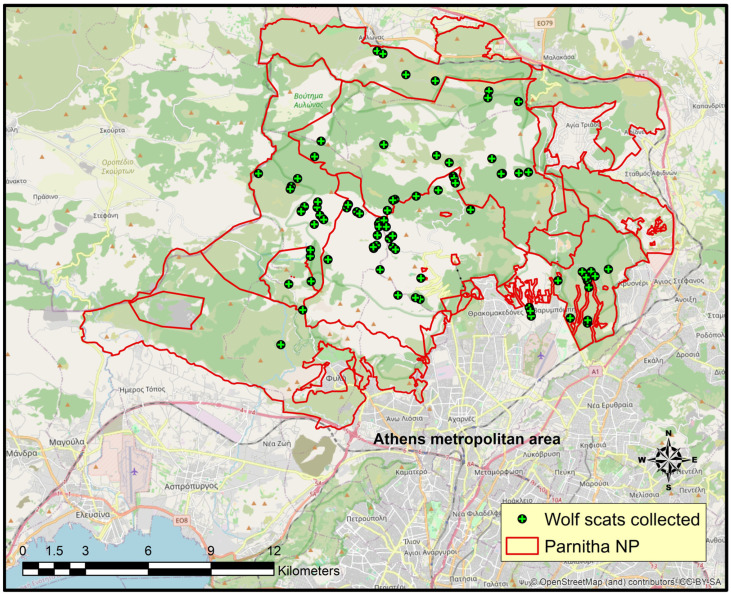
Distribution of wolf scat samples (green circles) collected from the Parnitha Protected Area.

Non-invasive genetic sampling was conducted from July 2022 to June 2023, covering a census area of approximately 200 km^2^ within the Parnitha Protected Area (Figure 1). This area includes a Special Area of Conservation (SAC) and a Special Protection Area (SPA) according to the Habitats (92/43/EEC) and Birds (2009/147/EC) Directives, respectively, coded as site GR3000001 in the Natura 2000 Network [20].

Epithelial tissue samples from fresh scats were collected mainly at intersections along dirt roads facilitated by recorded wolf presence by GPS satellite telemetry, camera trapping and field investigations of wolf bio-indices. The identification of wolf scats was carried out by examining their size, form, scent and contents [21]. Additionally, tissue samples were obtained from the genital tract of stray dogs as part of official sterilizations performed by local municipalities in the vicinity of the Parnitha area. Apart from the systematic sampling in the Parnitha area, a nationwide opportunistic collection of muscle tissue and blood samples from dead wolves that were morphologically identified as pure wolves was undertaken across the entire wolf distribution between 2010 and 2024 by the Callisto Wildlife and Nature Conservation Society. All samples were submerged in 99% denatured ethanol and stored at −20 °C.

### 2.2. DNA Extraction

DNA was extracted from 124 wolf scats, 50 wolf tissues and 50 dog tissues using the QIAamp Fast DNA Stool Mini Kit (Qiagen, Hilden, Germany) for scats, the QIAamp DNA Mini Kit (Qiagen, Hilden, Germany) for wolf tissues and the cetyltrimethylammonium bromide method [22] for dog tissues. DNA extraction efficiency from scat samples was evaluated through PCR amplification of a single locus, using multiple independent PCR replicates per sample, since the low concentration of obtained DNA does not allow direct testing on agarose gel. To verify the amplification of this locus, all PCR products were electrophorized on a 2% agarose gel.

### 2.3. PCR Amplification

Non-invasive samples were amplified in multiplex PCR at 20 canine-specific autosomal microsatellites: FH2079, FH2140 [23], C250, C253, C466 [24], CPH3, CPH4, CPH5, CPH6, CPH7, CPH8, CPH12 [25], vWF [26], FH3210, FH3241, FH2004, FH2658, FH4012, REN214L11 and FH2361 [27]. Wolf and dog tissue samples were also genotyped in 20 microsatellite loci (FH3210, FH3241, FH2004, FH2658, FH4012, REN214L11, FH2361), with multiplex amplification implemented similarly. These genotypes provided reference genetic profiles for comparison of allele frequencies at each locus to ascertain whether each scat sample belonged to a wolf, dog, or hybrid. The total volume of each reaction was 10 μL, which contained 0.1 μL of each primer and 1 μL of template DNA corresponding to 50–1000 ng from tissue and blood samples or 4 μL of scat DNA analogous to 20–40 ng. Amplifications were carried out using 2× Multiplex PCR Master Mix, 5× Q-Solution (Qiagen, Germany), and touchdown thermal cycling was started with denaturation at 94 °C for 3 min, followed by 10 cycles of 94 °C for 30 s, 65 °C for 1 min (decreasing 0.5 °C per cycle) and 72 °C for 30 s, and was continued for a further 30 cycles of 94 °C for 30 s, 60 °C for 1 min, and 72 °C for 30 s, with a final extension at 72 °C for 5 min.

All samples that showed positive PCR amplification during DNA quality assessment were subsequently genotyped using a multiple-tube approach at the 20 aforementioned microsatellite loci [28]. Two independent PCRs per locus were initially performed for each sample following the protocol of Adams and Waits [29]. An allele was accepted only if it was amplified at least twice; otherwise, a third PCR was performed. If amplification failed even after the third PCR, additional replicates were performed. If the allele was not amplified at least twice after up to five PCR replicates in total, the genotype at that locus was discarded. A negative control (without DNA) and a positive control (with known genotypes) were included in each PCR to detect contamination and confirm that the reaction worked, respectively.

The unique scat samples were amplified in three replicates using the Taq PCR Core Kit (Qiagen) for sex identification of two DNA fragments: a 106 bp fragment within the coding region of the sex-determining region Y (SRY) gene and a 185 bp fragment within the androgen receptor (AR) gene on the X chromosome of *Canis l.* [30]. Each 10 μL reaction consisted of 0.1 μL of each primer designed by van Asch et al. [30], 4 μL of genomic DNA (corresponding to approximately 20–40 ng), 0.75 units/μL of Qiagen Taq polymerase, 0.25 mM dNTPs, and 1 μL of 10× Qiagen PCR Buffer. The PCR program had an initial denaturation at 95 °C for 15 min; 35 cycles of 94 °C for 30 s, 60 °C for 90 s, and 72 °C for 1 min; and a final extension at 72 °C for 30 min. Following amplification, 2 μL of each PCR product was electrophoresed alongside a 100 bp ladder on a 2% agarose gel. When the amplification was tested with wolf tissue samples of known sex, it produced both fragments in a male and one 185 bp fragment in a female.

### 2.4. Data Analysis

All PCR products, except those used for sex identification, were analyzed on an ABI 3500 Genetic Analyzer with GeneScan 500 LIZ Dye Size Standard (Thermo Fisher Scientific, Waltham, MA, USA) and genotyped using Geneious 10.2.6 software [31]. Genotypes from 20 microsatellites were processed with DropOut 2.0 [32] to distinguish the unique individuals from recaptures (identical genotypes) in the studied population. The effectiveness of each microsatellite marker in distinguishing the unique individuals was assessed by the probability of identity (P_ID_ and P_ID-sibs_ [33]) in DropOut. Additionally, the cumulative probability of identity for all microsatellite loci was estimated using Cervus 3.0.7 [34]. The same software was used to estimate the frequency of null alleles (F_null_) per locus, while Micro-Checker 2.2 was employed to verify their existence. The number of alleles (N_A_), the observed (H_o_), the expected (H_e_) heterozygosity and the polymorphism information content (PIC) were calculated for each locus and the population using the Microsoft Excel add-in Microsatellite toolkit 3.1.1 [35]. Departure from the Hardy–Weinberg equilibrium (HWE) was assessed using Fisher’s method, with exact *p*-values derived from the Markov chain method implemented in Genepop 4.0.7 [36]. The inbreeding coefficient (F_IS_) was evaluated for each locus and the population using FSTAT v2.9.3 [37]. The effective population size (N_e_) was estimated using the linkage disequilibrium method (LD) applied in NeESTIMATOR 2.1 [38] as well as the sibship method implemented in COLONY 2.0.7.1 [39]. Principal coordinate analysis (PCoA) was implemented in GenAlEx 6.5 [40] to assess the occurrence of an underlying genetic structure.

The capwire package of R 4.3.3 was employed to estimate the census population size (N_c_), as it efficiently analyzes non-invasive genetic data from a single sampling session with multiple recaptures of individuals of small populations, such as the one studied [41]. Capwire uses two models for population estimation: the equal capture model (ECM), which assumes equal capture probability among individuals, and the two-innate rates model (TIRM), which accounts for heterogeneity by grouping individuals into two classes with different capture probabilities. A likelihood ratio test (LRT) was implemented in capwire to evaluate model fit.

The full-likelihood method was implemented in COLONY 2.0.7.1 [39] for sibship and parentage assignments, analyzing genotypes inferred from all 20 microsatellite loci. The mating system was defined as polygamous for both sexes, assuming no inbreeding, as recommended by the software developer when the inbreeding level is not high, in order to reduce the complexity of the method. To ensure the identification of all potential relationships, the analysis was performed twice. At first, all uniquely genotyped wolves were set as both offspring and potential parents. Subsequently, all distinct individuals were defined as offspring, while males and females were assigned as candidate parents. The probability of a father or mother being included in the male or female candidates, respectively, was assumed to be 0.5, since all breeding individuals were likely not sampled—for example, due to moderate sampling effort or dispersal. Additionally, genetic relatedness (r) between all pairs of distinct individuals was estimated using point estimates from seven estimators implemented in COANCESTRY 1.0.1.11 [42] as a complementary approach to verify kinship identified by COLONY. Inbreeding was not considered in this analysis. Since no prior data on known kinship were available for simulation, estimated pairwise relatedness values between 0.4 and 0.6 were mainly examined across all estimators, given that relatedness between parent–offspring and full siblings is typically around 0.5.

To detect potential wolf–dog hybrids in the studied population, a Bayesian clustering approach was applied using STRUCTURE 2.3.4 [43] assuming an admixture model with correlated allele frequencies of wolves and stray dogs from the study area, as well as reference wolves distributed across Greece. Ten independent runs were performed for each value of K ranging from 1 to 3 with 100,000 Markov Chain Monte Carlo (MCMC) iterations after a burn-in period of 10,000 iterations. The optimum K was determined using the Evanno ΔK method [44]. An admixture score threshold of 0.8 was set [45].

### 2.5. SNP-Based Testing of Wolf–Dog Hybrids

Harmoinen et al. [46] developed a reduced Single Nucleotide Polymorphism (SNP) panel comprising 93 ancestry informative markers for wolves and dogs, derived from the Illumina CanineHD Whole-Genome BeadChip (174 K). This panel was specifically designed for non-invasive samples and was selected for its high discriminative power between wolves and dogs and their hybrids up to the third backcross generation.

To assess the accuracy of the microsatellite analysis for hybrid identification, 30 high-quality non-invasive DNA samples from wolves in the Parnitha area were genotyped using this 93-SNP panel with the Standard BioTool v.9.3 (formerly Fluidigm Corp., San Francisco, CA, USA) microfluid array technology, tested on microfluidic 96.96 Dynamic Arrays^TM^ (Standard BioTools Inc., South San Francisco, CA, USA) [47]. A multiplexed pre-amplification step, known as specific target amplification (STA), with an increased DNA volume and STA-PCR cycles, was employed to enhance the amplification rate according to the improved protocol for SNP genotyping, as demonstrated in von Thaden et al. [47]. NewHybrids 1.1 Beta 3 software [48] was applied to the SNP dataset to assign samples to recent hybrid categories—wolf, dog, F1, and F2—and the two recent backcross generations to wolf or dog, respectively, with a burn-in of 250,000 steps, followed by 250,000 sweeps. For correct assignment, 20 samples, each from different European wolf populations—Central European (CEP), Baltic (BALT), Dinaric-Balkan (DIN) and Carpathian (CARP)—and 20 reference dogs from various common breeds, excluding wolf–dog breeds, were used. The use of genetically identified wolf references from different European populations has been recommended to account for the potential impact of population structure within European wolves that may bias assignment probabilities [8].

## 3. Results

### 3.1. Genotyping Success

Of the 124 scat samples collected from the area, only 57 samples (46%) passed the initial quality control (amplified in at least one locus) and were further amplified at the remaining loci. Forty-six samples (37%) were almost fully genotyped, and they were further analyzed. The mean allelic dropout rate was 2.3%, while the mean false allele rate was 0.3%. The mean polymorphism information content (PIC) was 0.55. The cumulative probability of identity (P_ID_ = 6.7 × 10^−15^ and P_ID-sibs_ = 7.3 × 10^−7^) was considerably low, ensuring that identical genotypes were recaptures of the same individual rather than distinct individuals sharing the same genotype by chance.

### 3.2. Population Size and Sex Ratio Estimates

From 46 genotyped samples, 31 distinct wolf individuals were identified, providing a minimum wolf population count. Twenty-two wolves (71%) were captured once, whereas nine wolves (29%) were captured between two and four times, with a mean of 1.42 captures per individual. The estimated N_c_ using the ECM was 57 wolves (95% CI: 40–67), while TIRM yielded a higher estimate of 76 individuals (95% CI: 56–100). The performance of the two models was compared using the LRT, with ECM providing a better fit (LR = 8.79, *p*-value = 0.18 > 0.1). The estimated N_e_ based on the linkage disequilibrium method was 12.5 (95% CI: 10.7–14.6), while that based on the sibship method was 9 (95% CI: 6–10.2). Sex was successfully determined for 65% of the unique individuals, with nine identified as males and 13 as females, resulting in a sex ratio of 0.69.

### 3.3. Genetic Diversity

All loci analyzed were polymorphic, with a mean of 4.35 alleles per locus (Table S1). The mean observed heterozygosity was 0.55, whereas the mean expected heterozygosity was 0.62. The entire dataset showed a significant deviation from HWE (multi-locus test), with a positive mean F_IS_ value (0.11), which reflected a deficit of heterozygotes. Single-locus tests detected deviations from the HWE at half of the microsatellite loci, while only CPH4 and C466 significantly deviated due to the excess of heterozygotes. Micro-Checker detected potential null alleles at two loci, which were the only ones with inflated F_null_ values (vWF: 0.23, FH2361: 0.25).

### 3.4. Sibship and Parentage Assignment

**Figure 2:**
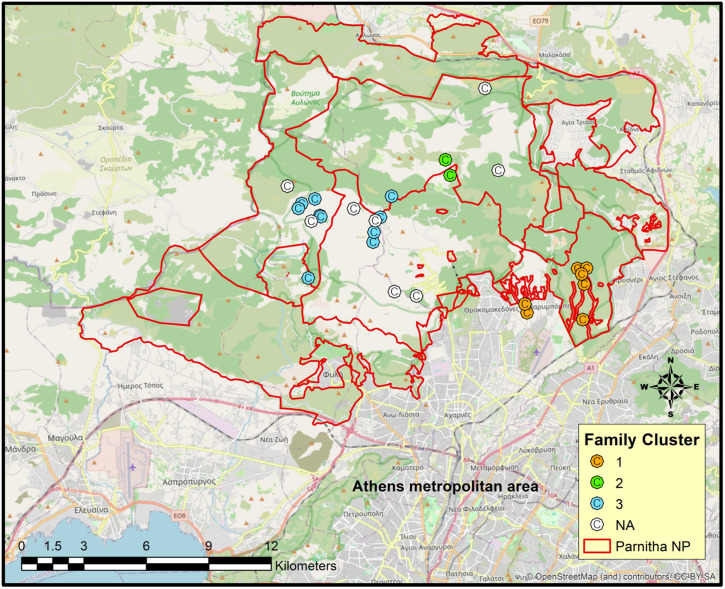
Distribution of wolf packs in the Parnitha Protected Area. Each dot represents a unique individual. Individuals not assigned to any group are shown in white (NA).

Pedigree analysis using COLONY revealed the presence of three distinct family groups within the study area (Figure 2). The primary family unit was detected in the southeastern part and consisted of a dominant breeding pair, O5 (male) and O6 (female), along with their offspring: O1, O13, O15, O16 and O23. The second family group was identified in the central part of the study area and consisted of a parent–offspring pair, with O10 assigned as the father of O22. Parent–offspring and full-sibling relationships in these two groups were likewise supported by relatedness estimates (r = 0.4–0.6). Another family group was also found in the core study area including eleven individuals (O3, O4, O7, O8, O18, O19, O24, O25, O26, O27, and O28). Among these, only one parent–offspring pair (O8 as the mother of O19) was detected. Individuals in this extended family unit were considered first- to third-degree relatives (i.e., parent–offspring, full-siblings, half-siblings, grandparent–grandchild, and avuncular and first cousins) based on pairwise relatedness values (r = 0.1–0.6) obtained from seven estimators using COANCESTRY. Eight individuals were not assigned to any group, as no parental or full-sibling relationship was found with any member of the defined groups. Three individuals were excluded prior to analysis because they were genotyped at 13 out of 20 loci, which could mask proper pedigree assignment.

### 3.5. Wolf–Dog Hybridization

**Figure 3:**
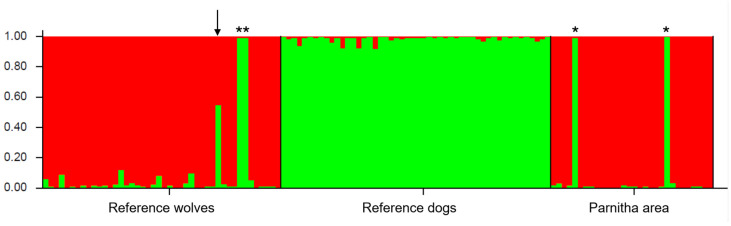
Bar plot from Bayesian cluster analysis implemented in STRUCTURE, showing individual genetic assignment to two inferred clusters (K = 2). Each color corresponds to a cluster, and each vertical bar corresponds to a single individual, with color proportions indicating the probability of assignment (0–1) to each cluster. Individuals with admixture scores of q ≥ 0.8 were classified as parental (wolf or dog), whereas those with q < 0.8 were considered admixed. Two individuals from the Parnitha area and two individuals among the reference wolves, denoted with asterisks, were identified as dogs, while one individual from the reference wolf samples, denoted with an arrow, turned out to be a potential F1 hybrid.

Forty-five of the 50 reference wolf tissue samples opportunistically collected over the last 15 years from across Greece and all 50 reference dog tissue samples collected from Mount Parnitha were successfully genotyped to perform Bayesian STRUCTURE analysis. According to the Evanno ΔK method, the entire dataset was best represented by two inferred genetic clusters (K = 2; Figure 3). All reference dogs were assigned to a single cluster (d), with a mean membership proportion of Q_d_ = 0.98 (q_d_ range = 0.921–0.995). From the unique individuals identified in the Parnitha Protected Area, 31 were assigned to cluster w (Q_w_ = 0.92, q_w_ range = 0.884–0.995), whereas two revealed dog ancestry with a membership proportion nearly equal to one (q_d_ = 0.991 and 0.995), indicating that both samples belonged to dogs, but they had initially been misidentified as wolf samples. The same result was obtained among the 45 reference wolves, where two individuals also turned out to be dogs. Of particular importance was the presence of one individual in the reference wolf dataset that was partially assigned to both clusters based on microsatellites, with an individual proportion of admixture of q_w_ = 0.451 and q_d_ = 0.549, providing strong evidence of a wolf–dog hybrid in Greece that probably belongs to the F1 generation [46].

In order to test the efficacy of the microsatellite loci used in the present study to detect wolf–dog hybrids, the reduced SNP panel designed by Harmoinen et al. [46] for non-invasive samples was applied only in the Parnitha samples. Thus, based on the 93-SNP panel, two of the 30 non-invasive Parnitha samples could not be successfully genotyped, and they were therefore excluded from further analysis. Of the 28 successfully genotyped samples, 27 were clustered as pure wolves, while one appeared to be a second-generation backcross to dog, although it could not be excluded that it was a purebred dog (Figure S3).

## 4. Discussion

In this study, microsatellite genotyping was performed to evaluate the status of wolves that recolonized Mount Parnitha, Central Greece, by determining possible family organization, identifying potential wolf–dog hybrids due to the proximity to an area with high human population density and estimating key demographic parameters. Our findings enhance the understanding of the conservation status of wolves present in Europe’s southernmost part.

### 4.1. Kin Relationships

Pedigree analysis is a valuable tool for wolf conservation, helping to track genetic lineage, identify inbreeding, and assess the effectiveness of management strategies. Pedigrees showed that wolves were structured in at least three distinct family units within the study area. The permanent presence of three packs—one in the southeastern edge and two in the core study area—has already been confirmed by the detection of wolf pups in both areas using camera trapping [49]. In contrast, relationships within the extended family group appeared unclear, reflecting a more fragmented structure. Field observations suggested that this region may function as an overlap zone and a territorial marking zone between two different wolf territories, as indicated by the high density of wolf scats. Recent wolf telemetry data from 2025 also support this hypothesis. Furthermore, this part of Parnitha hosts the highest density of red deer, which represents almost 67% of the biomass consumed by wolves in the Parnitha area [49]. These may explain the increased wolf presence in the central sector, even though many individuals were not closely related. It seems that within a short period, wolves successfully formed reproductive units in the Parnitha area, and they cannot just be considered a population sink consisting mostly of dispersing loners.

### 4.2. Hybrid Identification

The presence of wolves in the proximity of the Athens metropolitan area raises additional concerns regarding potential hybridization with domestic dogs. The risk of wolf–dog hybridization is likely more pronounced in habitats where a small wolf population or few recolonizing wolf dispersers coexist with a large population of free-ranging dogs, especially under intense human pressure like poaching that destroys pack structure [50].

In the case of the Parnitha area, Bayesian cluster analysis revealed no admixed wolves among the 31 distinct individuals detected in the area. Given that a substantially larger set of microsatellite markers is required to reliably detect backcrossed individuals, a conservative admixture score threshold was applied in the Bayesian cluster analysis to minimize the risk of misclassifying pure parental individuals as hybrids [45]. Individuals with admixture scores of q ≥ 0.8 were classified as parental (wolf or dog), whereas those with lower scores were considered admixed.

Furthermore, to validate the absence of hybrids in the Parnitha wolf population, a 93-SNP panel was applied, which detected one backcrossed individual, classified as a second-generation backcross to dog (Figure S3). However, considering that intrinsic wolf content is present in various dog breeds [51], this individual was most likely a domestic dog, a result that is also strongly supported by microsatellite analysis. No other hybrids were found in the studied population in the Parnitha Protected Area based on SNP analysis, thereby confirming the results of the microsatellite analysis and supporting the absence of recent hybridization events in the area. The absence of hybrids could be related to the area’s strict protection, which limits the presence of free-ranging dogs and human disturbance. As a result, wolves do maintain a stable social structure and feed predominantly on wild ungulates, with red deer and wild boar accounting for 67% and 14% of the consumed biomass, respectively [49]. Such conditions minimize encounters and breeding opportunities with free-ranging dogs, despite their presence in the surrounding areas, thus reducing the likelihood of hybridization [46].

Although no hybrids were detected in the Parnitha area, one possible wolf–dog hybrid was detected in the Greek wolf reference population that consisted of individuals spread across Greece. This individual was located in Northern Greece, in an area with locally higher densities of free-ranging dogs and a less strict protection status, which may be associated with more frequent hybridization events. This observation is further supported by camera-trap records showing that four out of 11 detected wolf packs included individuals with atypical morphological traits [52]. However, we should note here that there is a chance of false hybrid identification if an immigrant from a neighboring population is not represented in the wolf reference populations [53]. Including reference samples from other Dinaric–Balkan wolf populations in the future could help to avoid misidentifying immigrants as having dog ancestry.

### 4.3. Wolf Population Size in the Parnitha Area

Estimating wolf population size is crucial since it helps in understanding predator–prey relationships. Especially in the case of the Parnitha area, where red deer is considered critically endangered and the wolf population size affects red deer population dynamics, understanding this relationship is vital for the management of both species. The wolf census population size was estimated to be 57 individuals (95% CI: 40–67) using ECM or 76 individuals (95% CI: 56–100) using TIRM. Regardless of the model used, wolf population size and density are considered extremely high. The reasons are related to the conditions under which wolves recolonized Mount Parnitha. The reappearance of the species after a 60-year absence coincided with a period (2011–2014) when the red deer population exploded as a consequence of the wildfires in 2007, which created a more suitable grazing habitat for red deer. The red deer minimum population was estimated at 1000 individuals [17]; thus, there is a very high density of naïve-to-predation individuals. At the same time, the area is well protected, and the poaching of wolves is rare. The combination of abundant food sources (red deer, wild boar, and domestic animals) and the lack of systematic poaching quickly led to the formation of large packs, with a suitable pack size for hunting large prey. On the other hand, the absence of human-induced mortality maintained this population structure and density. Even though the Parnitha Protected Area is very close to the capital city of Greece, it essentially forms a natural system where the wolf population size is regulated based on the available food biomass.

We should mention here that there are technical issues that can improve accuracy in population size estimation. First, the mean recapture rate was below the minimum recommended threshold, which ensures reliable and precise estimates of population size [41]. One factor that can affect the recapture rate is the sampling season. In order to achieve an average of more than two captures per individual, field surveys should be intensified during autumn, winter, and early spring, when temperatures are lower than those in summer [54]. On the other hand, the preservation method is crucial for improving PCR amplification success and genotyping accuracy rates, which can ultimately help to increase recapture rates [55]. Except for high-concentration ethanol, routinely used to preserve wolf scat samples [29,56,57,58], an alternative storage medium such as DET’s buffer, which minimizes the enzymatic degradation of nuclear DNA [59], could result in higher PCR amplification success. Furthermore, the accuracy of genetic CR estimates may also be improved by increasing sampling effort [54], for example, by collecting 2.5–3 times as many samples as the assumed number of individuals in the examined population [60]. In this case, it is recommended that a preliminary population size estimation should be carried out—prior to implementing genetic CR methods—using alternative, lower-cost non-invasive methods such as camera trapping for minimum population size count or in combination with random encounter models (REM) [61].

### 4.4. Levels of Genetic Diversity

Genetic diversity, as measured by observed and expected heterozygosity, is a key indicator—along with population size—of a population’s evolutionary potential, allowing adaptation to environmental challenges. Low heterozygosity is often associated with bottlenecks or founder effects in small or isolated populations, potentially increasing the risk of inbreeding and reducing population fitness [62]. The relatively low heterozygosity values, as well as effective population values observed in the Parnitha wolf population, can be explained by the small number of individuals settled in the Parnitha area and subsequently organized into a few packs [18,19]. Low levels of genetic diversity have also been reported in other recolonized wolf populations experiencing the founder effect [57,63]. These parameters will be valuable for understanding long-term demographic processes only when additional monitoring data become available systematically.

Family structure can affect both heterozygosity values as well as HWE, and this was also the case in the present study. In most reported wolf studies, there is a general pattern of deviations from HWE [57,58,64]. This pattern is commonly attributed to the Wahlund effect, when hidden substructures exist within a population, such as family units in the case of wolves [64]. The Wahlund effect can result in an excess of homozygotes and a positive F_IS_, which may be misinterpreted as a sign of assortative breeding if no further analysis is performed [65]. To investigate whether the clear deviation from HWE resulted from the pack structure, a Bayesian STRUCTURE analysis (Figure S1) and a PCoA (Figure S2) were performed, both of which indicated the existence of two genetically distinct subpopulations. Subpopulation A, located at the southeastern edge of the study area, was entirely composed of seven individuals, all of whom were first-degree relatives, whereas subpopulation B consisted of 21 first- to third-degree relatives. When HWE was tested within each subpopulation, no significant deviation was found in subpopulation A, and only 30% of the microsatellite loci deviated in subpopulation B. Additionally, the mean H_o_ and H_e_ did not differ significantly in either subpopulation (A: H_o_ = 0.52, H_e_ = 0.46, *p*-value = 0.084; B: H_o_ = H_e_ = 0.57, *p*-value = 0.9; two-tailed *t*-test, α = 0.05). The pairwise F_ST_ value of 0.17 indicated high genetic differentiation between the two subpopulations, which further supports the hypothesis that the heterozygote deficit at the population level was likely due to the Wahlund effect.

## 5. Conclusions

This study provides the first genetic insights into the recently established wolf population in the Parnitha Protected Area. A minimum population size of 31 wolves, organized in at least three packs, was identified. A more rigorous genetic sampling is necessary to understand the social structure of such a high wolf-density area. The lack of hybrids may indicate that wolf–dog hybridization does not necessarily happen in recently recolonized, peri-urban environments in Southern Europe, which is an encouraging finding and pronounces the importance of well-managed protected areas as genetic strongholds of the wolf population. Future research needs to verify this through the inclusion of more sampling areas in protected vs. human-dominated areas. On the other hand, the identification of one possible F1 wolf–dog hybrid in Northern Greece underlines the need for further investigation of admixture events, especially in areas with high levels of individuals with atypical morphological traits, as denoted by a recent study in the Italian Peninsula where hybridization events reach almost 50% [66]. Continued non-invasive genetic monitoring, combined with standard field methods, is critical for detecting demographic shifts and early potential signs of hybridization. These efforts inform the development of effective strategies in response to the growing challenges of human–wolf coexistence in peri-urban areas.

## Data Availability

Data are available in Table S2 (Excel file) in the Supplementary Materials.

## Supplementary Materials

The following supporting information can be downloaded at: https://www.mdpi.com/article/10.3390/genes17030278/s1, Table S1: Summary statistics for the 20 microsatellite loci analyzed in this study. The following values are provided for each locus: number of alleles (N_A_), observed heterozygosity (H_o_), expected heterozygosity (H_e_), polymorphism information content (PIC), *p*-value for Hardy–Weinberg equilibrium exact test (HW; * *p* < 0.05 significant deviation), frequency of null alleles (F_null_), inbreeding coefficient (F_IS_), probability of identity (P_ID_), and probability of identity among siblings (P_ID-sibs_); mean values across loci are shown in the bottom row; Table S2: Multilocus genotypes of unique wolf individuals in the Parnitha Protected Area at 20 microsatellite loci; Figure S1: Bar plot showing individual assignment probabilities to two genetic clusters (K = 2) based on Bayesian STRUCTURE analysis. Each vertical bar represents an individual from the Parnitha wolf population. The proportions of red and green within each bar indicate the estimated individual ancestry from each of the two inferred genetic clusters; Figure S2: Scatter plot from Principal Coordinates Analysis (PCoA) based on microsatellite genotypes of 28 wolves from the Parnitha area. The plot illustrates genetic distances among individuals, with two distinguishable clusters corresponding to two distinct subpopulations (subpopulation A: right, subpopulation B: left). The first two principal coordinates individually explain 21.72% and 11.47% of the variance, while together they explain 33.19% of the variance; Figure S3: Posterior probability distribution estimated with NewHybrids software, showing reference wolf samples from different European populations: Central European (CEP), Baltic (BALT), Dinaric-Balkan (DIN) and Carpathian (CARP); reference dog samples from various common breeds, excluding wolf–dog breeds; and wolf samples from the Parnitha area assigned to recent hybrid categories: wolf, dog, F1, F2, and the two recent backcross generations to wolf or dog, respectively. Each vertical bar corresponds to an individual, and each color corresponds to a category. The height of each colored segment is proportional to the probability of that individual belonging to a certain category.
