# Supplementary material for: Genetic Monitoring of a Newly Established Grey Wolf Population in a Peri-Urban Protected Area with First Insights into Wolf–Dog Hybridization in Greece
Source: Genes (Basel). 2026 Feb 27;17(3):278. doi: 10.3390/genes17030278 (PMC13026283; doi:10.3390/genes17030278)
Supplement: Supplementary file 1 [file genes-17-00278-s001.zip › Supplementary_materials.pdf]

Supplementary tables and figures

Table S1. Summary statistics for the 20 microsatellite loci analyzed in this study. The following values are provided for each locus: number of alleles (N_A), observed heterozygosity (H_o), expected heterozygosity (H_e), polymorphism information content (PIC), p-value for Hardy-Weinberg equilibrium exact test (HW; *p<0.05 significant deviation), frequency of null alleles (F_{null}), inbreeding coefficient (F_{IS}), probability of identity (P_{ID}), and probability of identity among siblings ($P_{ID-sibs}$); mean values across loci are shown in the bottom row.

Locus	N_A	H_o	H_e	PIC	HW	F_{null}	F_{IS}	P_{ID}	$P_{ID-sibs}$
FH2079	2	0.29	0.38	0.30	0.30	0.14	0.26	4.61E-01	6.78E-01
CPH4	3	0.79	0.64	0.56	0.02*	-0.13	-0.23	9.68E-02	3.31E-01
C253	4	0.75	0.68	0.60	0.17	-0.06	-0.10	1.71E-02	1.51E-01
FH2140	6	0.64	0.72	0.66	0.01*	0.02	0.11	2.25E-03	6.53E-02
CPH5	2	0.29	0.25	0.21	1.00	-0.07	-0.15	1.35E-03	5.07E-02
CPH12	3	0.61	0.61	0.53	0.61	0.00	0.01	3.14E-04	2.57E-02
CPH3	4	0.54	0.65	0.56	0.61	0.09	0.18	6.51E-05	1.24E-02
C466	5	0.50	0.49	0.45	0.04*	0.01	-0.01	1.93E-05	7.24E-03
CPH6	7	0.67	0.78	0.73	0.00*	0.05	0.15	1.71E-06	2.81E-03
CPH7	4	0.63	0.74	0.68	0.02*	0.08	0.15	2.13E-07	1.17E-03
C250	5	0.71	0.78	0.73	0.00*	0.03	0.09	1.90E-08	4.56E-04

CPH8	5	0.68	0.71	0.64	0.60	0.01	0.05	2.81E-09	1.99E-04
vWF	4	0.43	0.67	0.61	0.00*	0.23	0.37	4.74E-10	9.19E-05
FH4012	4	0.62	0.66	0.58	0.56	0.03	0.07	8.87E-11	4.35E-05
FH2361	4	0.32	0.54	0.46	0.00*	0.25	0.41	2.53E-11	2.43E-05
REN214L11	3	0.54	0.44	0.35	0.55	-0.11	-0.22	1.02E-11	1.54E-05
FH3210	7	0.63	0.80	0.75	0.00*	0.10	0.21	8.17E-13	5.85E-06
FH3241	4	0.52	0.64	0.56	0.12	0.11	0.19	1.71E-13	2.87E-06
FH2004	8	0.61	0.77	0.72	0.00*	0.11	0.21	1.62E-14	1.14E-06
FH2658	3	0.36	0.43	0.34	0.54	0.07	0.16	6.70E-15	7.32E-07
Mean	4.35	0.55	0.62	0.55	0*		0.11	6.70E-15	7.30E-07

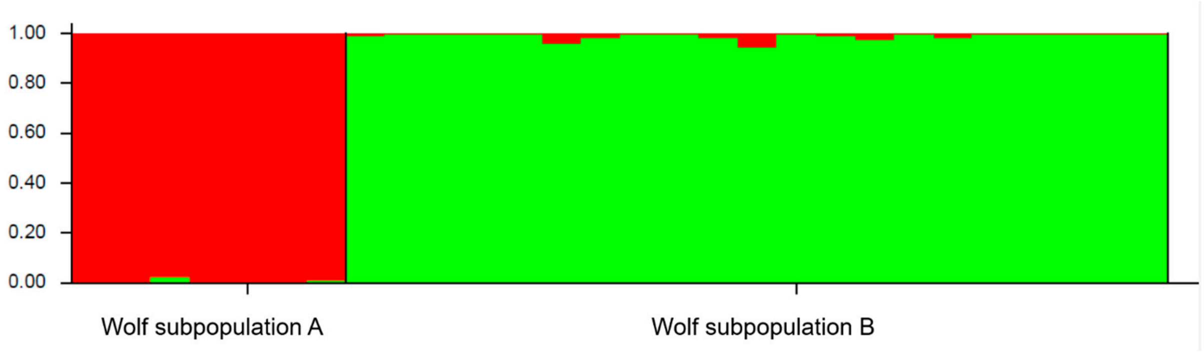

Figure S1. Bar plot showing individual assignment probabilities to two genetic clusters (K=2) based on Bayesian STRUCTURE analysis. Each vertical bar represents an individual from the Parnitha wolf population. The proportions of red and green within each bar indicate the estimated individual ancestry from each of the two inferred genetic clusters.

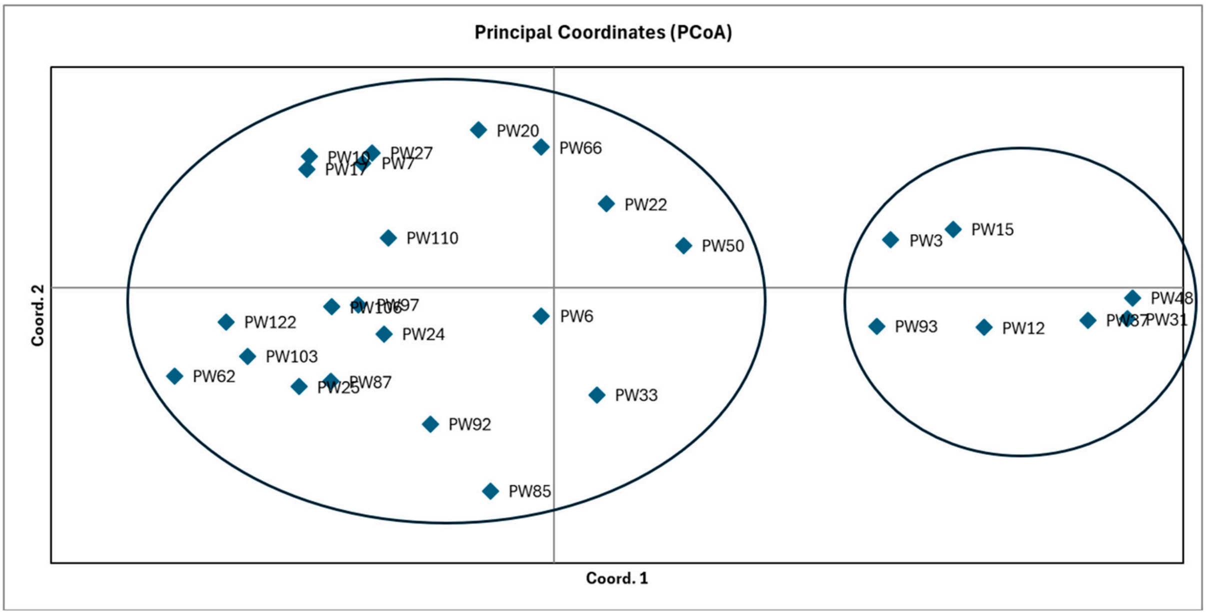

Figure S2. Scatter plot from Principal Coordinates Analysis (PCoA) based on microsatellite genotypes of 28 wolves from Parnitha area. The plot illustrates genetic distances among individuals, with two distinguishable clusters corresponding to two distinct subpopulations (subpopulation A: right, subpopulation B: left). The first two principal coordinates individually explain 21.72% and 11.47% of the variance, while together they explain 33.19% of the variance.

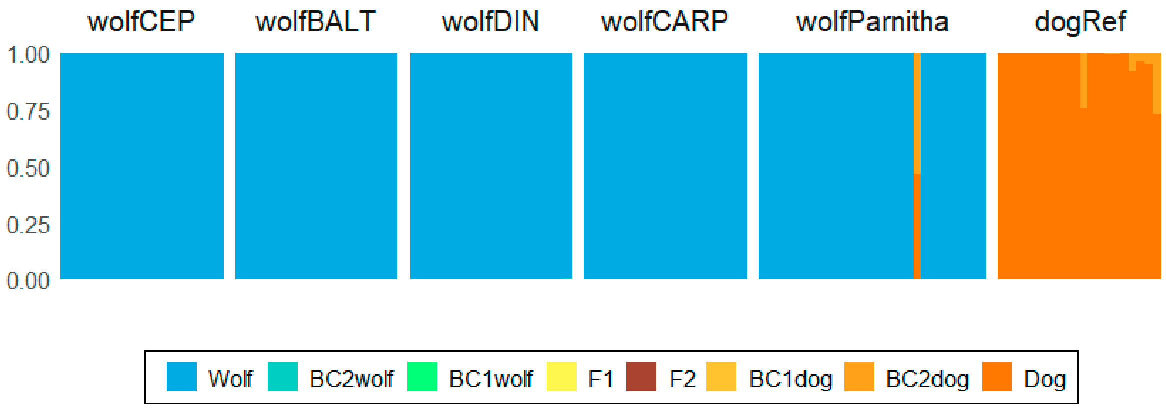

Figure S3. Posterior probability distribution estimated with NEWHYBRIDS software, showing reference wolf samples from different European populations: central European (CEP), Baltic (BALT), Dinaric-Balkan (DIN) and Carpathian (CARP); reference dog samples from various common breeds, excluding wolf-dog breeds; and wolf samples from the Parnitha area assigned to recent hybrid categories–wolf, dog, F1, F2, and the two recent backcross generations to wolf or dog, respectively. Each vertical bar corresponds to an individual, and each color corresponds to a category. The height of each colored segment is proportional to the probability of that individual belonging to a certain category.
